# Correction: Novel phenoxyacetic herbicides synthesized from longifolene-derived primary amine for sustainable weed management

**DOI:** 10.1039/d5ra90130h

**Published:** 2025-11-24

**Authors:** Yanqun Huang, Pingping Lu, Hongyun Lan, Daozhan Huang, Yu Feng, Fengguo Ya, Ziqi Gao, Jiaxin Wen, Ziqiang Zhao

**Affiliations:** a School of Materials and Environment, Guangxi Minzu University Nanning 530105 China; b Key Laboratory of Chemistry and Engineering of Forest Products, State Ethnic Affairs Commission, Guangxi Key Laboratory of Chemistry and Engineering of Forest Products, Engineering Research Center of Low-carbon and High-quality Utilization of Forest Biomass, University of Guangxi, School of Chemistry and Chemical Engineering, Guangxi Minzu University Nanning 530006 China huangdaozhan@gxmzu.edu.cn

## Abstract

Correction for ‘Novel phenoxyacetic herbicides synthesized from longifolene-derived primary amine for sustainable weed management’ by Yanqun Huang *et al.*, *RSC Adv.*, 2025, **15**, 38251–38259, https://doi.org/10.1039/D5RA05630F.

The authors regret that the original article contains an error in affiliation *a*. The correct affiliation is as displayed herein.

^*a*^School of Materials and Environment, Guangxi Minzu University, Nanning 530105.

The authors also regret an error in the Graphical Abstract of the original article. The correct version of the Graphical Abstract is shown below.
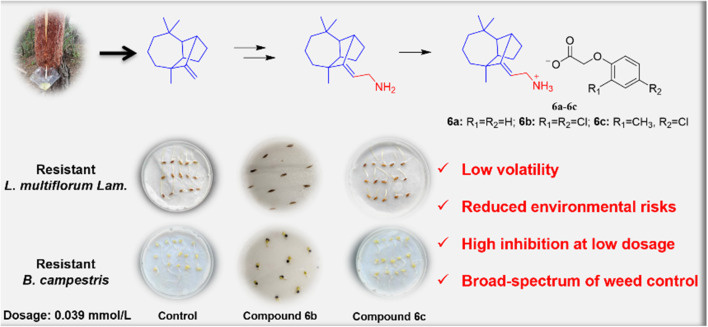


The authors also regret an error in Scheme 1 of the original article. The R_1_ substituent of compound **6c** is CH_3_ instead of H. The correct reaction scheme is shown below.

**Scheme 1:**
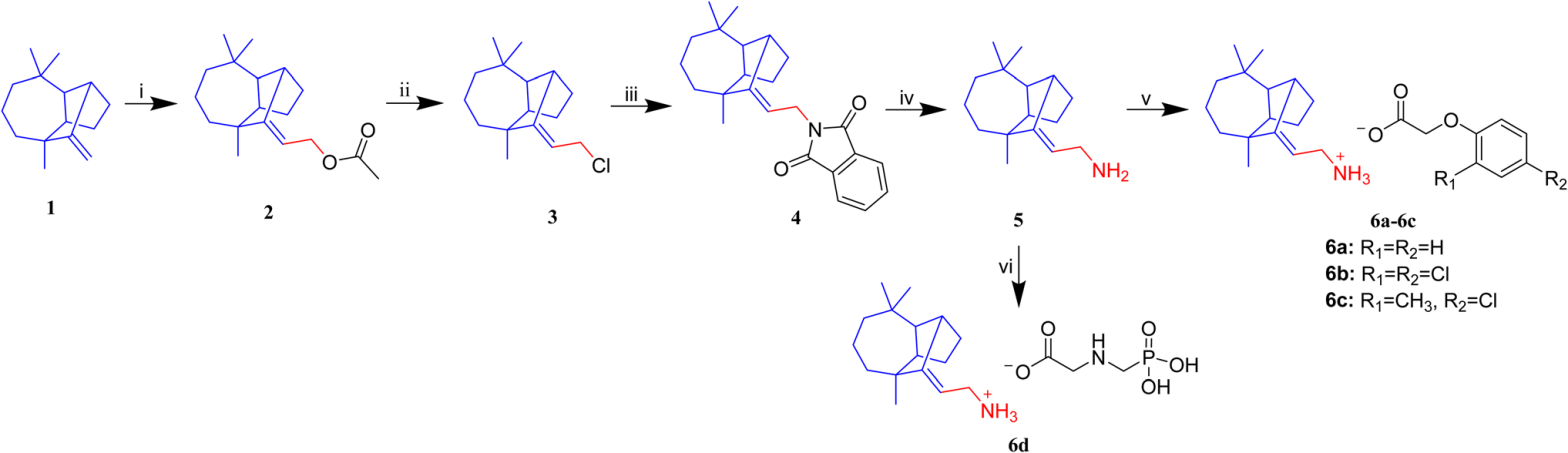
Synthetic route of target compounds **6a–6d** (i) CH_3_COOH, (HCHO)_*n*_, reflux, 24 h; (ii) CH_3_COCl, reflux, 1 h; (iii) potassium phthalimide, DMF, 2 h; (iv) N_2_H_4_·H_2_O, NaOH, reflux, 6 h; (v) absolute alcohol, 80 °C, 1.5 h; (vi) glyphosate, deionized water, RT, 30 min.

Finally, the authors also regret an error in the description of the comparison of the IC_50_ value of compound **6d** against *Lolium multiflorum* Lam. with that of GLYP-IPAM salt appearing in lines 10–11, page 38255 of the original article. The correct version is:

“The IC_50_ value of compound **6d** against *Lolium multiflorum* Lam. root growth was higher than that of GLYP-IPAM salt, but lower than GLYP-IPAM salt against *Lolium multiflorum* Lam. shoot growth.”

The Royal Society of Chemistry apologises for these errors and any consequent inconvenience to authors and readers.

